# Validity and reliability of the Arabic sedentary behavior questionnaire among university students aged between 18–30 years old

**DOI:** 10.1186/s12889-023-15030-1

**Published:** 2023-01-18

**Authors:** Mohammad A. Alahmadi, Khalid H. Almasoud, Amani H. Aljahani, Naweed S. Alzaman, Omar M. Al Nozha, Osama M. Alahmadi, Rola A. Jalloun, Eman M. Alfadhli, Jomana M. Alahmadi, Areeg A. Zuair, Naif S. Alzahrani, Ahmed A. Alahmdi, Mansour A. Alghamdi, Bachir Zoudji, Abdulaziz A. Aldayel, Nasser M. Al-Daghri

**Affiliations:** 1 Physical Education and Sport Sciences Department, Taibah University, Medina, Saudi Arabia; 2 Physical Sport Science Department, Princess Nourah Bint Abdulrahman University, Riyadh, Saudi Arabia; 3 Internal Medicine Department, Taibah University, Medina, Saudi Arabia; 4 College of Medicine, Dar Al Uloom University, Riyadh, Saudi Arabia; 5 Nutrition and Food Science Department, Taibah University, Medina, Saudi Arabia; 6 Medicine Department, Taibah University, Medina, Saudi Arabia; 7 College of Pharmacy, King Saud University, Riyadh, Saudi Arabia; 8 Community Health Nursing Department, Taibah University, Medina, Saudi Arabia; 9 Medical Surgical Nursing Department, Taibah University, Medina, Saudi Arabia; 10 College of Medicine, Al-Rayan Colleges, Medina, Saudi Arabia; 11 Anatomy Department, King Khalid University, Abha, Saudi Arabia; 12 Visual and Urban Design Laboratory, University Polytechnique Hautes-de-France, Valenciennes, France; 13 Exercise Physiology Department, King Saud University, Riyadh, Saudi Arabia; 14 Biomarkers of Chronic Diseases, Biochemistry Department, College of Science, King Saud University, Riyadh, Saudi Arabia

**Keywords:** Arabic SBQ, Sedentary behavior, IPAQ-SF, Validity

## Abstract

**Purpose:**

The study aimed to test the validity and reliability of the Arabic version of the sedentary behavior questionnaire (SBQ).

**Methods:**

A total of 624 university students (273 males; 351 females, mean age = 20.8 years) were recruited from Taibah University, Madinah, Saudi Arabia. For criterion and constructive validity (*n* = 352), the Arabic SBQ was compared with total sitting time from the International Physical Activity Questionnaire-short form (IPAQ-SF) and the International Physical Activity Questionnaire-long form (IPAQ-LF). For concurrent validity, the English and Arabic SBQ versions were given concurrently to bilingual university students (*n* = 122) once. For test–retest reliability, the Arabic SBQ was given twice to participants (*n* = 150) at a one-week interval.

**Results:**

Sitting time of IPAQ-SF (7^th^ question: sitting time on weekdays) and IPAQ-LF (21^st^ question: sitting time on weekdays and 22^nd^ question: sitting time on weekends) correlated significantly with total sitting time/week of the Arabic SBQ (*r* = 0.29, p = 0.003; *r* = 0.14, *p* = 0.02, respectively). Motorized transportation measured with the IPAQ-LF correlated significantly with time spent driving in a car, bus, or train from the Arabic SBQ on weekdays and weekends (*r* = 0.53, *p* < 0.001; *r* = 0.44 *p* < 0.001, respectively). The total sitting time of the Arabic SBQ was inversely correlated with BMI (*r* = -0.18, *p* = 0.001). The correlations between the Arabic and the English SBQ versions ranged from 0.25–0.96; *p* < 0.001 on weekdays and 0.50–0.90; *p* < 0.001 on weekends. Moderate to good reliability was also found between test and retest for all SBQ items and total score during weekdays (0.72 to 0.8), and weekends (0.64 to 0.87), with exception of the 7^th^ item "play musical instrument", ICC = 0.46). Mean difference of test–retest of the Arabic SBQ was not significantly different from zero for the total sitting time of the Arabic SBQ (t = -0.715, *P* = 0.476).

**Conclusion:**

The Arabic SBQ had satisfactory levels of reliability, with total sitting time of the Arabic SBQ correlating significantly with sitting times derived from IPAQ-SF, IPAQ-LF, and the English SBQ versions. Hence, the Arabic SBQ can be used as a tool to measure sedentary behavior among adult Arabs aged between 18 to 30 years old in future epidemiologic and clinical practice.

**Supplementary Information:**

The online version contains supplementary material available at 10.1186/s12889-023-15030-1.

## Background

Sedentary behaviour is an attribute that has gained accumulating evidence to be separate from an overlapping characteristic known as physical inactivity [1–4]. It has become increasingly clear that sedentary behaviour differs from not doing physical activity or not meeting recommended levels of moderate-to-vigorous physical activity [5, 6].

A recent meta-analysis study showed that sedentary behaviour is more likely to be underestimated if few items were used in questionnaires, compared to a multi-domain questionnaire such as the Sedentary Behavior Questionnaire (SBQ) [7, 8]. SBQ is designed to obtain detailed estimates of sedentary behaviour as it includes 9 behavioral types (watching television, playing computer/video games, sitting while listening to music, sitting and talking on the phone, doing paperwork or office work, sitting and reading, playing a musical instrument, doing arts and crafts, and sitting and driving/riding in a car, bus or train on weekdays and weekends) [7, 8]. SBQ as a validated questionnaire adapted and used in different language versions such as Spanish [9, 10], Turkish [11], Slovenian [12], German and Danish [10]. Since sedentary behaviour is particularly common in modern urban life, attention has been paid to its prevalence with respect to other ethnic groups such as the Saudi Arabian people, and, in fact, for the first time, recommendations regarding sedentary behavior prevention was introduced (i.e. The 24-h Movement Practice Guidelines for Saudi Arabia) [13]. Few researches have been conducted in Saudi Arabia on sedentary behaviour, relying on a questionnaire focusing on a single domain, such as screen time (watching TV, using the internet, and playing electronic games) [14–19]. To the best of our knowledge, no version of the original English SBQ has been established in Arab countries. Therefore, this study aimed to adapt the English version of SBQ to the Saudi population and test its validity and reproducibility on a sample of students aged between 18 to 30 years old in Madinah, Western Saudi Arabia.

## Methods

### Participants and study design

A total of 624 university students [43.8% male (*n* = 273); 56.3% females (*n* = 351)] aged between 18 and 30 years old participated in this cross-sectional study. All participants were from Taibah University in Madinah, Saudi Arabia. University students were recruited from different colleges (i.e. College of Pharmacy, College of Education, College of Dentistry, College of Medical Rehabilitation Sciences, College of Art and Humanities, College of Applied Medical Sciences, College of Engineering, College of Science, College of Medicine, and College of Nursing) during the second semester of the academic year of 2022. Information such as age, gender, height, and weight were self-reported and obtained from the participants.

**Fig. 1:**
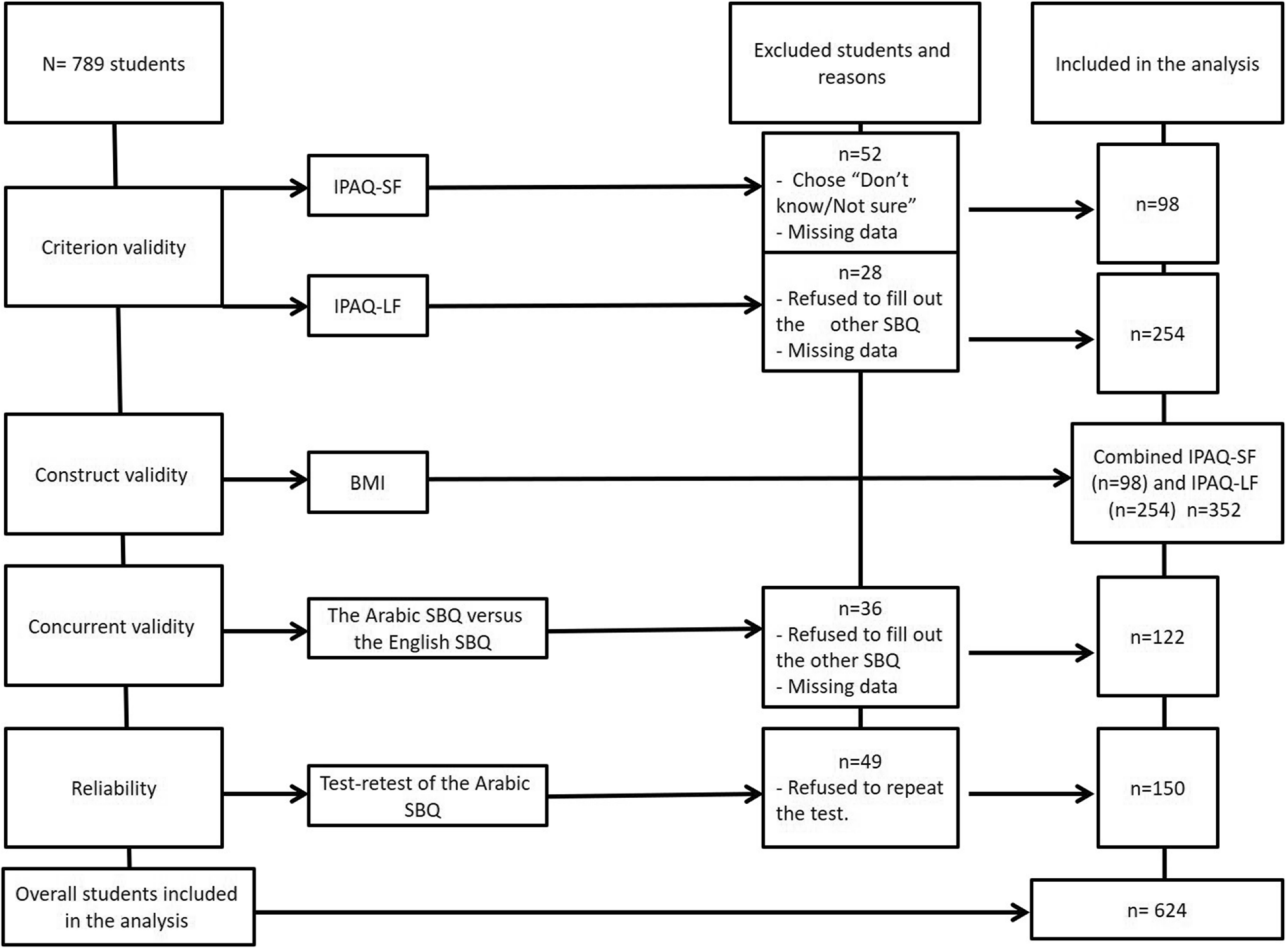
A flowchart of sample stages of the study

The sample size was based on the respondent-to-item ratio which ranged from 5:1 (i.e., 50 participants for a 10-item questionnaire) to 10:1 (i.e., 100 participants for a 10-item questionnaire) [20]. Therefore, based on the number of items in all questionnaires used in our study, the required sample size was from 105 to 210 participants for criterion and constructive validity, 90 to 180 participants for concurrent validity, and 90 to 180 participants for test retest reliability. We added more participants because it has been suggested to add approximately 15% to the sample size as required for each parametric test [21]. Increasing the sample size is important because it avoids any expected loss of data such as withdrawals from the study or missing data [21]. We started with 789 university students and ended with 624 students (criterion and constructive validity: *n* = 352; concurrent validity, *n* = 122; test retest reliability, *n* = 150). Therefore, a total number of 624 university students, who answered all items of SBQ, were included in this study (see Fig. 1).

### Translation process

SBQ was adopted from Rosenberg et al. [8] and included nine behavioral forms on weekdays and weekends (1. watching television, 2. playing computer/video games, 3. sitting while listening to music, 4. sitting and talking on the phone, 5. doing paperwork or office work, 6. sitting and reading, 7. playing a musical instrument, 8. doing arts and crafts, and 9. sitting and driving/riding in a car, bus or train) (see Appendix 1).

The translation process was carried out in stages according to guidelines for the cross-cultural adaption process recommended by Beaton et al. [22]. The first stage was that the original English SBQ was forward translated into Arabic by two bilingual translators (T1 and T2). The goal at this stage was to establish semantic equivalence between the English and Arabic versions. The second stage was the synthesis of the translation where the expert committee (i.e. translators, healthcare specialists, methodologists, exercise physiology experts, and a language professional) assessed the translated questionnaire to ensure that the Arabic-translated version was idiomatically and conceptually equivalent. The two translators (T1 and T2) synthesized the results of the translation and generated the first common translation (T-12) for the Arabic SBQ version. The third stage is the back translation where the Arabic SBQ version (T-12) was blindly back-translated by two English-speaking translators who had no prior knowledge of the original English version. This is regarded as checking validity for inconsistency or conceptual errors that occurred due to translation. In the fourth stage, the expert committee reviewed the back-translated English questionnaire to the original English questionnaire in order to identify discrepancies and resolve any contradictions between the two versions. The forward-back translation procedure was repeated until a consensus was reached. A pilot study was conducted on 38 participants who were excluded in the main analysis of the present study. The pre-final questionnaire was used to assess its application and comprehension. Based on the outcomes of the pilot study, a minor change was made due to cultural adaptation. The 4^th^ item of the SBQ, "sitting and talking on the phone" was changed to “sitting and talking on the phone or the mobile phone” because "the phone" alone in the Arabic language can be referred to as a landline phone, so we added "the mobile phone" to the 4^th^ item. The Arabic SBQ's final version was approved to be used in the current study.

### Criterion and constructive validity

The Arabic SBQ was compared with reported total sitting time from the International Physical Activity Questionnaire-short form (IPAQ-SF) and International Physical Activity Questionnaire-long form (IPAQ-LF). Both forms of IPAQ were already validated in the Arabic language [23] and so the sitting time of both IPAQ-SF and IPAQ-LF were used to support validity of the current study. The amount of time reported sitting in the original long and short IPAQ forms are already valid [24]. IPAQ-SF has one item about sitting time, (item #7, during the last 7 days, how much time did you usually spend sitting on weekdays?). This item was compared to the items of the Arabic SBQ (during weekdays and weekends). The total sitting time of the Arabic SBQ (min/week) was calculated as follows: [(sedentary behavior on weekdays × 5) + (sedentary behavior on weekends × 2)]. IPAQ-LF also has two items about sitting time on weekdays and weekends (item #26, during the last 7 days, how much time did you spend sitting on a weekday?" and item number 27 "During the last 7 days, how much time did you usually spend sitting on a weekend day?). The total sitting time of IPAQ-LF (min/week) was calculated as follows: [(sitting time on weekdays × 5) + (sitting time on weekends × 2)]. This procedure used to score the IPAQ-LF is available on the IPAQ website (www.ipaq.ki.se). We compared sitting time on weekdays and weekends measured with IPAQ-LF with the items of the Arabic SBQ (during weekdays and weekends). We also compared the total sitting time of IPAQ-LF with the total sitting time of the Arabic SBQ. Moreover, IPAQ-LF has one additional item regarding sitting time in motorized transportation. We compared the item of transportation measured by IPAQ-LF with the 9^th^ item "time spent driving in a car, bus, or train" from the Arabic SBQ on weekdays and weekends. Finally, construct validity with body mass index (BMI) was used in the original English version of SBQ [8]. Therefore, for constructive validity, we compared BMI with the items of the Arabic SBQ and the total sitting time of SBQ.

### Concurrent validity

Items of the Arabic SBQ were compared with the original English SBQ version for concurrent validity. Both versions were administered consecutively during the same interview to bilingual students (*n* = 122) from Medical and Health Colleges at Taibah University.

### Reliability of the Arabic SBQ

For the test–retest reliability study, the Arabic SBQ was given twice to the participants (*n* = 150) one week apart. In order to ensure a higher response rate, all participants completed the IPAQ-SF, IPAQ-LF, the English SBQ version and the Arabic SBQ version during a face-to-face interview with the researchers or assistant researchers.

### Statistical analysis

Statistical analyses were carried out using SPSS (Version 22). Data was expressed as mean ± standard deviations or 95% confidence interval (CI). For criterion validity, Pearson correlation analysis was used to evaluate the correlation between the items of the Arabic SBQ and the total sitting time of the Arabic SBQ with both IPAQ-SF and IPAQ-LF. A one-sample t-test was used to determine systematic bias in the mean difference between sitting time of the Arabic SBQ on weekdays (min/day) and sitting time of IPAQ-SF on weekdays (min/day). A one-sample t-test was also used to determine systematic bias in the mean difference between the total sitting time of the Arabic SBQ and the total sitting time derived from IPAQ-SF and IPAQ-LF. For constructive validity, Pearson correlation was used to evaluate the association between BMI and the items of the Arabic SBQ.

For concurrent validity, the items of the SBQ in both Arabic and English forms were compared using Pearson correlation analysis. Bland–Altman plot analysis was used to evaluate the extent of agreement between the total sitting time of the Arabic and the English versions of the SBQ [25]. Subsequently, a one-sample t-test was used to determine systematic bias in the mean difference between the total sitting time of the Arabic and the English versions of the SBQ.

For the reliability of the Arabic SBQ, the test–retest reliability of each item was assessed using the intra-class correlation coefficient (ICC) and Pearson’s correlation analysis. Bland–Altman plot analysis was also used to evaluate the extent of agreement between the total sitting time of the first and the second administration of the Arabic version of SBQ. Subsequently, a one-sample t-test was used to assess systematic bias on the mean difference between the total sitting time of the first and second Arabic SBQ. The ICC value was interpreted as follows: < 0.5 indicates poor, 0.5–0.75 indicates moderate, 0.75–0.9 indicates good, and > 0.9 indicates excellent reliability [26].

## Results

A total of 624 respondents answered all items of the SBQ and were hence included in the study [criterion and constructive validity, *n* = 352; concurrent validity, *n* = 122 participants; test–retest reliability *n* = 150). Table 1 shows the characteristics of respondents.

**Table 1:** Characteristics of students (273 males and 351 females)

Variables	Mean ± SD	Minimum	Maximum
Age (year)	20.82 ± 1.92	18	30
Height (cm)	164.15 ± 8.87	145	190
Weight (kg)	60.09 ± 14.73	37	130
BMI (kg/m^2^)	22.12 ± 4.15	13.63	42.45

*BMI* Body mass index

### Criterion and constructive validity

Descriptive data of criterion and constructive validity are presented in Table 2 while data on criterion and constructive validity are presented in Table 3. Low but significant correlations were found between the Arabic SBQ items, total sitting time, IPAQ-SF, IPAQ-LF, and BMI, ranging from *r* = -0.11 to *r* = 0.36 (Table 3). The results showed that the 7^th^ question (i.e., sitting time on weekdays) from IPAQ-SF significantly correlated with items one (i.e., TV), four (i.e., sit talk on the phone), and five (i.e., office/paper work) of the Arabic SBQ on weekdays and with items one (i.e., TV) three (i.e., sit listen to music) four (i.e., sit talk on the phone) and eight (i.e., arts and crafts) on weekends. Moreover, both the 7^th^ question (i.e., sitting time on weekdays) from IPAQ-SF and question 21^st^ (i.e., sitting time on weekdays) and 22^nd^ (i.e., sitting time on weekends) from IPAQ-LF correlated significantly with the total sitting time per week of the Arabic SBQ (*r* = 0.29, *p* = 0.003; *r* = 0.14, *p* = 0.02, respectively). Inverse correlations were found between BMI and the 5^th^ (i.e., office/paper work) and 6^th^ (i.e., reading) items of the Arabic SBQ on weekdays (*r* = -0.20, *p* < 0.001; *r* = -0.12, *p* = 0.02, respectively) and weekends (*r* = -0.14, *p* = 0.01; *r* = -0.11, *p* = 0.048, respectively), while the 4^th^ item (i.e., sit talk on the phone) was only significant on weekends (*r* = -0.13, *p* = 0.016). Total sitting time of the Arabic SBQ was also inversely correlated with BMI (*r* = -0.18, *p* = 0.001).

**Table 2:** Descriptive date (M ± SD) of The Arabic sedentary behavior questionnaire Items, Total sitting time of the Arabic SBQ, IPAQ-SF (*n* = 98), IPAQ-LF, (*n* = 383), and BMI (*n* = 481)

Items	Criterion validity				Constructive validity	
	**IPAQ-SF** (*n* = 98)		**IPAQ-LF**, (*n* = 383)		**BMI** (*n* = 481)	
	**Weekdays**	**Weekends**	**Weekdays**	**Weekends**	**Weekdays**	**Weekends**
1-TV (min/day)	63.65 ± 77.59	87.34 ± 94.67	52.91 ± 67.35	74.76 ± 85.95	55.88 ± 70.38	78.26 ± 88.51
2-Computer/games (min/day)	46.47 ± 67.72	68.97 ± 88.00	41.02 ± 73.15	58.93 ± 89.07	42.54 ± 71.62	61.73 ± 88.77
3-Sit listen to music (min/day)	37.44 ± 45.28	48.04 ± 51.19	37.97 ± 65.65	46.83 ± 70.35	37.82 ± 60.61	47.16 ± 65.54
4-Sit talk on the phone (min/day)	102.04 ± 95.03	121.63 ± 97.36	126.31 ± 108.44	150.59 ± 115.34	119.55 ± 105.31	142.52 ± 111.25
5-Office/paper work (min/day)	98.67 ± 100.38	65.82 ± 80.40	90.35 ± 105.6	52.97 ± 78.25	92.67 ± 104.10	56.52 ± 78.94
6-Reading (min/day)	33.92 ± 47.25	33.92 ± 50.06	47.00 ± 66.07	36.14 ± 56.26	43.36 ± 61.63	35.52 ± 54.54
7-Play musical instrument (min/day)	12.19 ± 26.78	10.20 ± 23.87	4.07 ± 16.23	3.80 ± 16.82	6.33 ± 20.03	5.60 ± 19.24
8-Arts and crafts (min/day)	21.90 ± 47.06	24.28 ± 38.53	16.41 ± 41.29	21.41 ± 52.96	17.93 ± 42.97	22.21 ± 49.33
9-Sitting driving/riding in a car, bus, or train (min/day)	63.46 ± 72.09	80.45 ± 84.61	99.56 ± 86.64	114.15 ± 97.96	89.51 ± 84.31	104.77 ± 95.52
Total sitting time of the 9 items of the Arabic SBQ (min/day)	478.92 ± 245.78	539.54 ± 265.83	515.49 ± 238.14	559.58 ± 244.73	505.31 ± 240.50	554.00 ± 250.56
Total sitting time of the Arabic SBQ (min/week)	3473.72 ± 1694.31		3696.62 ± 1598.44		3634.56 ± 1626.34	
Sitting time (IPAQ-SF) (min/day)	477.73 ± 260.74					
Sitting time (IPAQ-LF) (min/week)			2323.49 ± 1275.37			
Motorized transportation (IPAQ-LF) (min/day)			148.84 ± 142.26			

*BMI* Body mass index, *SBQ* Sedentary behavior questionnaire, *IPAQ-SF* International physical activity questionnaire-short form, *IPAQ-LF* International physical activity questionnaire-long form

**Table 3:** Validity Associations between Arabic sedentary behavior questionnaire Items, Total sitting time, IPAQ-SF (*n* = 98), IPAQ-LF, (*n* = 383), and BMI

Items	IPAQ-SF		IPAQ-LF			BMI		
	***R* Weekdays**	***r* Weekends**		***r* Weekdays**	***r* Weekends**		***r* Weekdays**	***r* Weekends**
1-TV (min/day)	0.36^**^	0.30^*^		0.26^**^	0.20^**^		-0.077	-0.087
2-Computer/games (min/day)	-0.19	-0.15		0.014	0.083		-0.036	-0.058
3-Sit listen to music (min/day)	0.18	0.23^*^		0.043	0.153^*^		-0.022	-0.027
4-Sit talk on the phone (min/day)	0.23^*^	0.30^*^		0.099	0.105		-0.066	-0.13^*^
5-Office/paper work (min/day)	0.28^*^	0.16		0.079	0.069		-0.20^**^	-0.14^*^
6-Reading (min/day)	0.00	0.04		0.012	0.007		-0.12^*^	-0.11^*^
7-Play musical instrument (min/day)	-0.11	-0.13		0.013	0.061		0.33	0.055
8-Arts and crafts (min/day)	-0.13	-0.25^*^		0.028	0.013		0.56	0.068
9-Sitting driving/riding in a car, bus, or train (min/day)	0.13	0.07		0.115	0.132*		0.022	-0.024
Total sitting time of the Arabic SBQ (min/week)	0.29^*^		0.14^*^			-0.18^**^		

*BMI* Body mass index, *SBQ* Sedentary behavior questionnaire, *IPAQ-SF* International physical activity questionnaire-short form, *IPAQ-LF* International physical activity questionnaire-long form *r* Pearson’s correlation coefficients

^*^p < 0.05

^**^p < 0.01

**Fig. 2:**
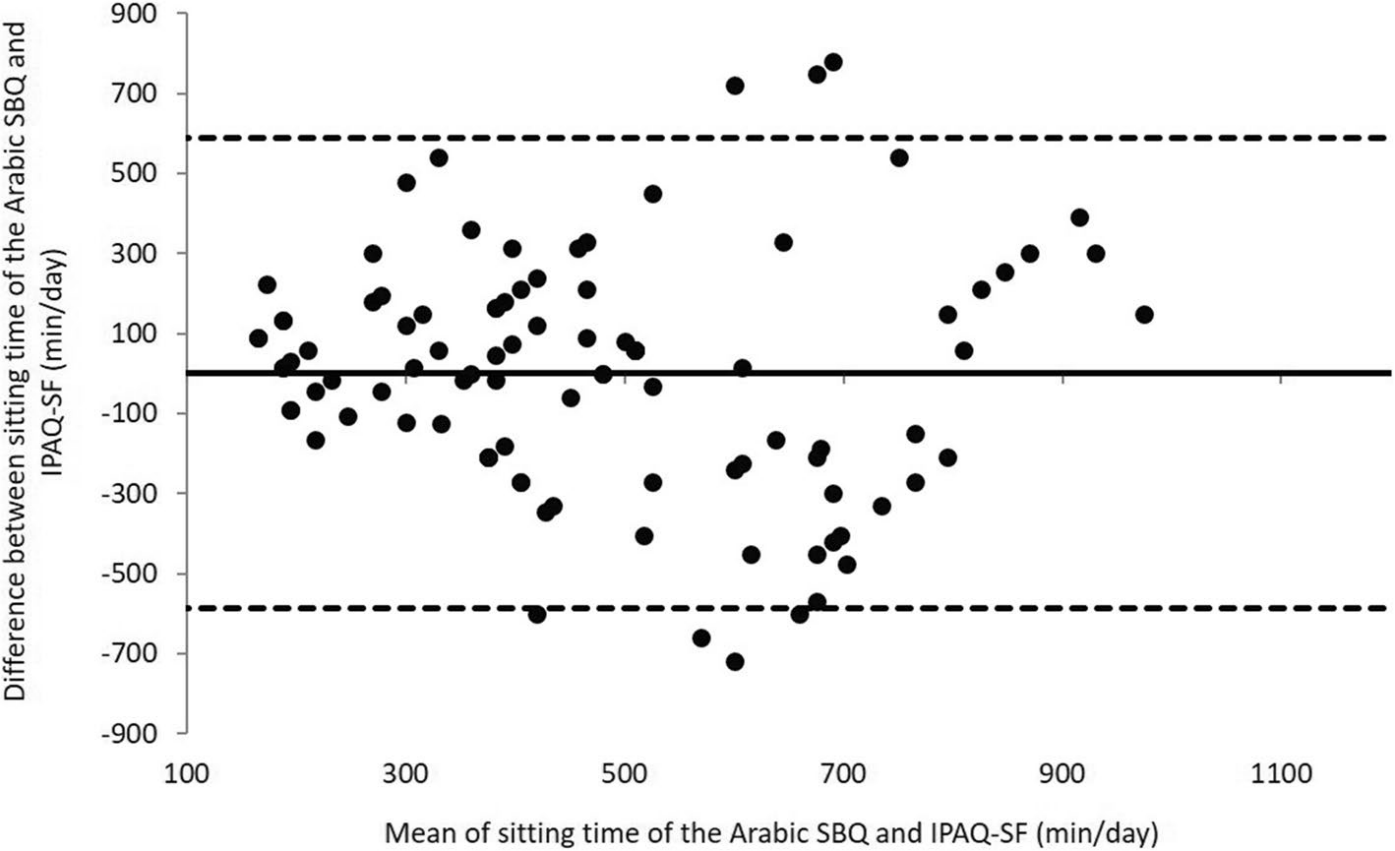
Bland Altman plot analysis of the Total sitting time (min/week) measured with the English and Arabic SBQs. Mean difference (solid line) = 35.94 min/week, 95% limits of agreement (ditched line) =  − 1420.2 to 1492.0

Figure 2 demonstrated a satisfactory agreement between the Arabic SBQ and IPAQ-SF for sitting time on weekdays (mean difference = 1.19 min/day, 95% limits of agreement =  − 586.68 to 589.07). A one-sample t-test also showed that the mean difference between sitting time of the Arabic SBQ on weekdays (min/day) and sitting time of IPAQ-SF on weekdays (min/day) was not significantly different from zero (t = 0.039, *P* = 0.969), indicating no systematic bias between the total sitting time of the Arabic SBQ and total sitting time of the IPAQ-LF (mean difference = 1.19 min/day).

A one-sample t-test showed that the mean difference between the Arabic SBQ and IPAQ-LF was significantly different from zero for total sitting time (t = 11.544, *P* = 0.000), indicating systematic bias between the total sitting time of the Arabic SBQ and total sitting time of the IPAQ-LF (mean difference = 1373.13 min/week). However, when we tested the association between the item of transportation measured by IPAQ-LF with the 9^th^ item (i.e., sitting driving in a car, bus, or train) from the Arabic SBQ on weekdays and weekends, the results of Pearson analysis showed a significant correlation between motorized transportation and item number nine from the Arabic SBQ on weekdays and weekends (*r* = 0.53, *p* < 0.001; *r* = 0.44 *p* < 0.001, respectively).

### Concurrent validity

Mean and ± SD of 9 items of both the Arabic and the English SBQ are shown in Table 4. The results of the concurrent validity are also presented in Table 4. A significant correlation was found between the Arabic and the English responses of bilingual university students regarding all of the SBQ’s items and total time spent sitting, with the exception of the item of playing a musical instrument and sitting driving/riding in a car, bus, or train during the weekends. The significant correlations between the Arabic and the English SBQ versions ranged from 0.25 to 0.96 on weekdays and from 0.50 to 0.90 on weekends. Figure 2 demonstrated a satisfactory agreement between the English and the Arabic SBQ for total sitting time (mean difference = 35.94 min/week, 95% limits of agreement =  − 1420.2 to 1492.0). The mean difference between the Arabic and the English SBQ versions was not different for total sitting time and showed no bias between both questionnaires.

**Table 4:** Validity of the Arabic SBQ versus the English SBQ using Pearson's correlation (*n* = 122)

Items	Arabic SBQ (M ± SD)		English SBQ (M ± SD)		*r*	*r*
	**Weekdays**	**Weekends**	**Weekdays**	**Weekends**	**Weekdays**	**Weekends**
1-TV (min/day)	73.03 ± 91.18	87.54 ± 99.11	74.38 ± 92.51	93.59 ± 100.13	0.96^**^	0.90^**^
2-Computer/games (min/day)	40.94 ± 72.38	58.01 ± 89.47	40.08 ± 72.47	65.08 ± 101.48	0.70^**^	0.79^**^
3-Sit listen to music (min/day)	57.54 ± 67.14	70.28 ± 82.15	55.57 ± 66.51	72.52 ± 78.23	0.89^**^	0.85^**^
4-Sit talk on the phone (min/day)	123.07 ± 124.27	139.71 ± 125.76	122.33 ± 120.09	136.37 ± 123.23	0.92^**^	0.90^**^
5-Office/paper work (min/day)	115.41 ± 122.19	86.87 ± 111.28	111.63 ± 120.83	95.08 ± 114.49	0.93^**^	0.84 ^**^
6-Reading (min/day)	43.15 ± 71.70	45.99 ± 80.95	44.50 ± 78.06	38.14 ± 66.61	0.83^**^	0.87 ^**^
7-Play musical instrument (min/day)	6.51 ± 19.94	5.08 ± 16.94	5.57 ± 24.58	6.69 ± 36.27	0.25^**^	0.50 ^**^
8-Arts and crafts (min/day)	15.49 ± 44.95	16.36 ± 38.09	14.26 ± 43.07	18.22 ± 44.63	0.91^**^	0.90^**^
9-Sitting driving/riding in a car, bus, or train (min/day)	62.21 ± 75.02	65.57 ± 79.79	66.64 ± 79.97	73.63 ± 89.35	0.85^**^	0.58 ^**^
Total sitting time of SBQ (min/week)	3823.03 ± 1919.34		3858.97 ± 1915.24		0.92^**^	

*SBQ* Sedentary behavior questionnaire, *r* Pearson’s correlation coefficients

^**^p < 0.001

### Reliability

Table 5 presents mean and ± SD of test–retest values of the Arabic SBQ items for weekdays and weekends. The coefficients ranged from 0.34 to 0.78 during weekdays and from 0.51 to 0.77 during weekends for the items of the Arabic SBQ. The r value of total sitting time for test and retest of the Arabic SBQ was 0.77, *p* = 0.001.

**Table 5:** Mean and ± SD of test–retest values of the Arabic SBQ items during weekdays and weekends (*n* = 268)

**Items**	Test		Retest	
	**Weekdays**	**Weekends**	**Weekdays**	**Weekends**
1-TV (min/day)	53.65 ± 67.60	77.41 ± 89.22	50.03 ± 69.54	74.49 ± 90.76
2-Computer/games (min/day)	37.95 ± 71.97	55.87 ± 84.85	43.72 ± 78.54	59.09 ± 83.68
3-Sit listen to music (min/day)	33.62 ± 61.09	38.45 ± 58.85	37.75 ± 65.44	45.60 ± 74.16
4-Sit talk on the phone (min/day)	119.79 ± 107.31	143.95 ± 113.46	115.67 ± 93.71	131.47 ± 108.80
5-Office/paper work (min/day)	88.52 ± 106.26	56.07 ± 81.77	96.51 ± 110.37	63.12 ± 81.45
6-Reading (min/day)	47.11 ± 68.73	33.82 ± 53.20	42.88 ± 65.33	45.90 ± 67.10
7-Play musical instrument (min/day)	3.42 ± 14.49	2.44 ± 12.16	5.63 ± 24.87	6.18 ± 27.15
8-Arts and crafts (min/day)	15.30 ± 41.75	20.53 ± 53.04	22.09 ± 47.31	22.14 ± 54.76
9-Sitting driving/riding in a car, bus, or train (min/day)	93.02 ± 88.32	99.96 ± 91.60	86.84 ± 78.20	94.12 ± 87.14
Total sitting time of the 9 items of the Arabic SBQ (min/day)	492.41 ± 236.90	528.52 ± 248.12	501.00 ± 237.03	542.11 ± 268.22
Total sitting time of the Arabic SBQ (min/week)	3519.12 ± 1600.91		3589.26 ± 1631.71	

*SBQ* Sedentary behavior questionnaire

Table 6 presents test–retest ICC values of the Arabic SBQ items for weekdays and weekends. With the exception of the 7^th^ item "play musical instrument" on weekdays, ICC = 0.46), moderate to good reliability was found between test and retest for all the Arabic SBQ items and the total score during weekdays (ranged from 0.72 to 0.87) and weekends (ranged from 0.64 to 0.87).

**Table 6:** Test–retest for the Arabic sedentary behavior questionnaire (*n* = 268)

Items	ICC (95% CI)		*r* (95% CI)	
	**Weekdays**	**Weekends**	**Weekdays**	**Weekends**
1-TV (min/day)	0.82^**^ (0.78–0.86)	0.85^**^ (0.79–0.89)	0.70^**^ (0.61–0.77)	0.74^**^ (0.66–0.80)
2-Computer/games (min/day)	0.86^**^ (0.80–0.89)	0.87^**^ (0.82–0.90)	0.75^**^ (0.68–0.81)	0.77^**^ (0.70–0.83)
3-Sit listen to music (min/day)	0.72^**^ (0.60–0.80)	0.66^**^ (0.53–0.75)	0.57^**^ (0.45–0.67)	0.51^**^ (0.38–0.62)
4-Sit talk on the phone (min/day)	0.81^**^ (0.73–0.86)	0.82^**^ (0.75–0.87)	0.68^**^ (0.59–0.76)	0.70^**^ (0.61–0.77)
5-Office/paper work (min/day)	0.83^**^ (0.77–0.88)	0.82^**^ (0.76–0.87)	0.71^**^ (0.63–0.78)	0.70^**^ (0.61–0.77)
6-Reading (min/day)	0.73^**^ (0.62–0.80)	0.79^**^ (0.71–0.85)	0.57^**^ (0.45–0.67)	0.67^**^ (0.57–0.75)
7-Play musical instrument (min/day)	0.46^**^ (0.25–0.61)	0.64^**^ (0.50–0.74)	0.34^**^ (0.19–0.47)	0.63^**^ (0.53–0.72)
8-Arts and crafts (min/day)	0.87^**^ (0.82–0.90)	0.85^**^ (0.80–0.90)	0.78^**^ (0.71–0.83)	0.75^**^ (0.67–0.81)
9-Sitting driving/riding in a car, bus, or train (min/day)	0.82^**^ (0.76–0.87)	0.77^**^ (0.68–0.83)	0.70^**^ (0.61–0.78)	0.62^**^ (0.51–0.71)
Total sitting time of the 9 items of SBQ (min/day)	0.86^**^ (0.80–0.89)	0.86^**^ (0.81–0.90)	0.75^**^ (0.67–0.81)	0.76^**^ (0.68–0.82)
Total sitting time of SBQ (min/week)	0.87^**^ (0.82–0.90)		0.77^**^ (0.69–0.83)	

*SBQ* Sedentary behavior questionnaire, *r* Pearson’s correlation coefficients, *CI* Confidence interval, *ICC* Intra-class correlation coefficient

^**^p < 0.001

**Fig. 3:**
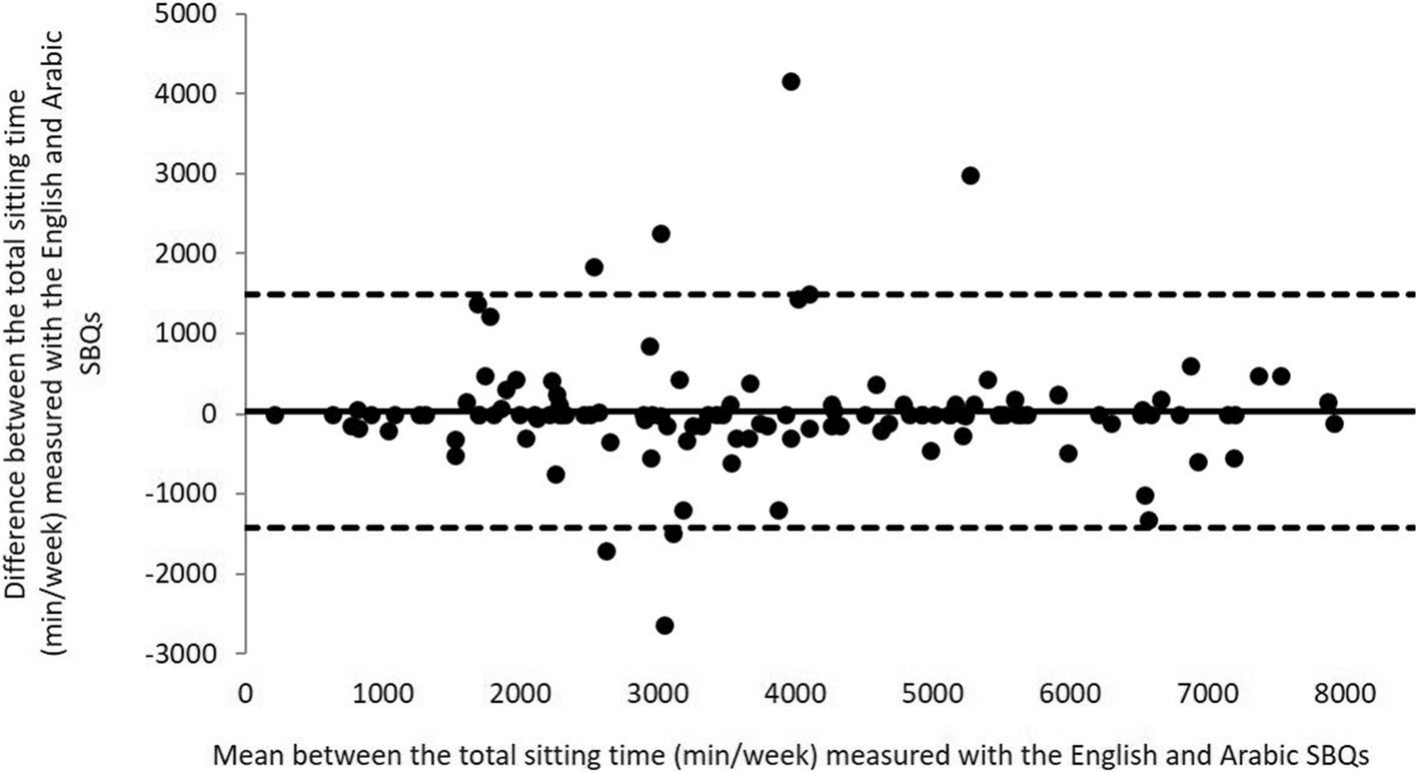
Bland–Altman plot analysis of the total sitting time of SBQ [mean difference (solid line) =  − 193.88 min/week, 95% limits of agreement (dashed line) =  − 2381.89; 1994.13]

**Fig. 4:**
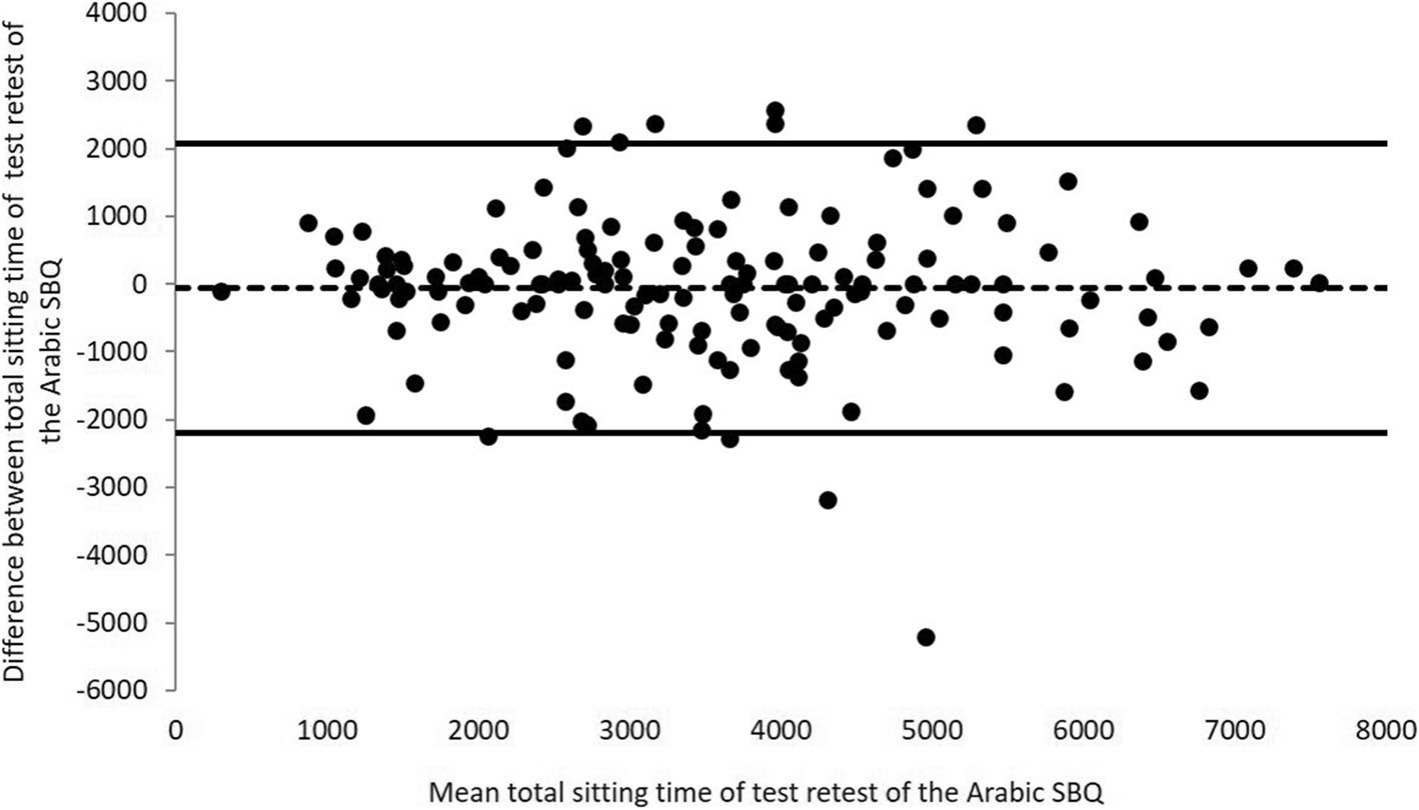
Bland–Altman plot analysis of the total sitting time of Arabic SBQ (t = -0.715, *P* = 0.476), indicating no bias between test–retest of the Arabic SBQ

Figure 3 shows the Bland–Altman plot analysis of the total sitting time of the Arabic SBQ (mean difference =  − 63.66 min/week, 95% limits of agreement =  − 2200.47; 2073.13). The mean difference between measurements was not different for the total sitting time of the Arabic SBQ (t = -0.715, *P* = 0.476), indicating no bias between test–retest of the Arabic SBQ (Fig. 4).

## Discussion

This study is the first to adapt the original English version of SBQ into the Arabic language, and to test the validity and reliability of the Arabic SBQ. The present study showed that the Arabic version of SBQ has moderate to good levels of reliability as a tool to assess sedentary behavior among the Saudi university students aged from 18 to 30 years old.

In terms of criterion-related validity, the current study compared the Arabic version of SBQ with two forms of the self-reported IPAQ. IPAQ-SF has a single item asking about sitting time during the last 7 days on weekdays, whereas IPAQ-LF has three items asking about sitting time during weekdays and weekends, and while driving or riding in a vehicle. There were two studies that used SBQ to compare its items with sitting time derived from both IPAQ-SF [11] and IPAQ-LF [8]. Bakar Y et al. [11] found a poor association between IPAQ-SF and most items of the Turkish version of SBQ, the highest correlation was with the 5^th^ item "Doing paperwork or computer work" on weekdays, *r* = 0.279 (*P* = 0.001). In our study, higher correlations were found on items such as item no 1 "TV" (*r* = 0.36, *p* < 0.001), item no 4 "Sit talk on the phone" (*r* = 0.30, *p* = 0.002), item no 5 "Office/paper work" (*r* = 0.28, *p* = 0.005), and total sitting time (*r* = 0.29, *p* = 0.003). Notably, the low correlations found between IPAQ-SF and the Arabic version of SBQ were expected with the 7^th^ item "Playing a musical instrument", *r* = -0.11, *p* = 0.24, and with the 9^th^ item "sitting driving or riding in a car, bus or train", *r* = 0.13, *p* = 0.18. Possible reasons for these low correlations may be because cars are regarded as the main type of transportation, and playing a musical instrument is also not a common activity in Saudi Arabia. Surprisingly, in the current study, the 4^th^ item "Sitting and talking on the phone" was correlated with only IPAQ-SF on weekdays (*r* = 0.23, *P* = 0.019) and weekends (*r* = 0.30, *P* = 0.002). This correlation of the 4^th^ item was not found with IPAQ-LF. In the Turkish version of SBQ, an association was found between this item and IPAQ-SF on weekdays (*r* = 0.24, *p* = 0.001) and on weekends (*r* = 0.17, *p* = 0.018). In fact, the 4^th^ item was changed to “sitting and talking on the phone or being busy on the phone” due to the extensive usage of smartphones nowadays. Although we explained this important point regarding this item to all participants during face-to-face interviews, it may be necessary to add "the usage of the phone" to the 4^th^ item of the Arabic SBQ. Of additional interest was that motorized transportation measured with the IPAQ-LF correlated item number nine (time spent driving in a car, bus, or train) from the Arabic SBQ on weekdays and weekends. In the original SBQ, Rosenberg et al., (2010) found a similar correlation between motorized transportation measured with the IPAQ-LF and time spent driving in a car, bus, or train from the SBQ (Partial *r* = 0.54, *P* = 0.01) [8].

The original English SBQ conducted construct validity and showed positive associations between BMI and total sitting time of SBQ in overweight adult males and females [8]. We found an inverse correlation BMI (*r* = -0.18, *p* = 0.001). It should be noted that the average BMI of our participants is within the normal age range (22.12 ± 4.15). This may explain why we had inverse correlations, compared to the original English SBQ. Interestingly, in the original English SBQ, BMI was correlated to the 1^st^ item "TV time" in overweight adults (*r* = 0.14 to 0.18) [8]. These contradictory results are not unexpected since watching TV is not a widespread type of sedentary behavior nowadays. It can be interpreted that the general Saudi community is generally sedentary and this was exacerbated during lockdowns [19].

Results from the Bland–Altman plot analysis give additional support and showed a satisfactory agreement between the English and the Arabic SBQ for total sitting time. A smaller mean difference found in our study (36 min/week, or 5 min/day) represents a small discrepancy between the two versions. Recently, a meta-analysis study showed underestimating sedentary behavior by sitting time questioners such as IPAQ-SF (-161.67), IPAQ-LF (-271.67), Global Physical Activity Questionnaire = (-219.85), with exception of the original SBQ which showed the best performance with a mean difference of -5.8 min/day [7].

Our study showed higher reliability between test and retest for all SBQ items and the total score during weekdays (ranged from 0.72 to 0.87) and weekends (ranged from 0.64 to 0.87), compared to the Turkish SBQ adaptation study (ICCs ranged from 0.40 to 0.72) [11]. Our findings were also similar to a Spanish SBQ adaptation study [9] and to the original English version [8]. In the present study, total sitting time had similar test–retest reliability for weekdays and weekends sedentary behaviors, indicating that time spent in different types of sedentary behaviors may not differ during weekdays and weekends. This finding is contrary to the finding from the original version of the SBQ, which reported that test–retest reliability was higher for weekday than weekend sedentary behaviors among overweight adults [8]. The previous research indicates that overweight/obese adults change their behaviors during weekends. For example, overweight/obese adults were found to spend more time watching TV on weekends compared to weekdays [27].

It is important to note that the mean difference between the first and second Arabic SBQ was not significantly different from zero for total sitting time. This good reliability may be because the preferred recommendations of sedentary behavior questionnaires were followed [28]. Additionally, the good reliability found in our study could be due to the fact that the questionnaire was administered by the researchers or the research assistants using a face-to-face interviewing method which is regarded as one of the strengths of the present study.

### Strengths and limitations

To the best of our knowledge, the current study is the first to adapt the SBQ to the Arabic language. We also determined a high correlation between answers of the English and Arabic SBQ version, and moderate to good test–retest reliability for most of the Arabic SBQ items. Finally, we provided a reliable multi-item questionnaire for Arabic-speaking countries to assess sedentary behavior. However, our study is not without shortcomings. The main limitation of our study is that we did not use device-assessed tools such as accelerometers or other devices to objectively assess sedentary behaviors. Therefore, the criterion validity evolution is highly recommended for future research to compare the Arabic SBQ to a gold-standard criterion measure. The data was collected only from one city, which is considered another limitation. Finally, the adults in the sample were university students and this may bias the current findings. Therefore, it is highly recommended to further evaluate the questionnaire in different populations of the Arab-speaking countries with different age groups.

## Conclusion

Based on our findings, the Arabic SBQ was found to be a reliable measure of sedentary behavior among Saudi university students. It is, therefore, available for use in future epidemiologic and clinical practice.

## Supplementary Information

**Additional file 1.** Appendix 1.

## Data Availability

The datasets generated and/or analyzed during the current study are available from the corresponding author on reasonable request.

## Abbreviations

SBQ: Sedentary behavior questionnaire
IPAQ-SF: International physical activity questionnaire-short form
IPAQ-LF: International physical activity questionnaire-long form
