# Supplementary material for: Validity and reliability of the Arabic sedentary behavior questionnaire among university students aged between 18–30 years old
Source: BMC Public Health. 2023 Jan 18;23:128. doi: 10.1186/s12889-023-15030-1 (PMC9847195; doi:10.1186/s12889-023-15030-1)
Supplement: Supplementary file 1 — Additional file 1. Appendix 1. [file 12889_2023_15030_MOESM1_ESM.docx]

**Appendix 1**

| **SEDENTARY BEHAVIOR: Weekday** | | | | | | | | | |
| --- | --- | --- | --- | --- | --- | --- | --- | --- | --- |
| **On a typical WEEKDAY, how much time do you spend (from when you wake up until you go to bed) doing the following?** | | | | | | | | | |
|  | **None** | **15**  **min. or less** | **30**  **min.** | **1**  **hr** | **2**  **hrs** | **3**  **hrs** | **4**  **hrs** | **5**  **hrs** | **6**  **hrs or more** |
| 1. Watching television (including videos on VCR/DVD). | 🞅 | 🞅 | 🞅 | 🞅 | 🞅 | 🞅 | 🞅 | 🞅 | 🞅 |
| 2. Playing computer or video games. | 🞅 | 🞅 | 🞅 | 🞅 | 🞅 | 🞅 | 🞅 | 🞅 | 🞅 |
| 3. Sitting listening to music on the radio, tapes, or CDs. | 🞅 | 🞅 | 🞅 | 🞅 | 🞅 | 🞅 | 🞅 | 🞅 | 🞅 |
| 4. Sitting and talking on the phone. | 🞅 | 🞅 | 🞅 | 🞅 | 🞅 | 🞅 | 🞅 | 🞅 | 🞅 |
| 5. Doing paperwork or computer work (office work, emails, paying bills, etc.) | 🞅 | 🞅 | 🞅 | 🞅 | 🞅 | 🞅 | 🞅 | 🞅 | 🞅 |
| 6. Sitting reading a book or magazine. | 🞅 | 🞅 | 🞅 | 🞅 | 🞅 | 🞅 | 🞅 | 🞅 | 🞅 |
| 7. Playing a musical instrument. | 🞅 | 🞅 | 🞅 | 🞅 | 🞅 | 🞅 | 🞅 | 🞅 | 🞅 |
| 8. Doing artwork or crafts. | 🞅 | 🞅 | 🞅 | 🞅 | 🞅 | 🞅 | 🞅 | 🞅 | 🞅 |
| 9. Sitting and driving in a car, bus, or train. | 🞅 | 🞅 | 🞅 | 🞅 | 🞅 | 🞅 | 🞅 | 🞅 | 🞅 |

| **SEDENTARY BEHAVIOR: Weekend Day** | | | | | | | | | |
| --- | --- | --- | --- | --- | --- | --- | --- | --- | --- |
| **On a typical WEEKEND DAY, how much time do you spend (from when you wake up until you go to bed) doing the following?** | | | | | | | | | |
|  | **None** | **15**  **min. or less** | **30**  **min** | **1**  **hr** | **2**  **hrs** | **3**  **hrs** | **4**  **hrs** | **5**  **hrs** | **6**  **hrs or more** |
| 1. Watching television (including videos on VCR/DVD). | 🞅 | 🞅 | 🞅 | 🞅 | 🞅 | 🞅 | 🞅 | 🞅 | 🞅 |
| 2. Playing computer or video games. | 🞅 | 🞅 | 🞅 | 🞅 | 🞅 | 🞅 | 🞅 | 🞅 | 🞅 |
| 3. Sitting listening to music on the radio, tapes, or CDs. | 🞅 | 🞅 | 🞅 | 🞅 | 🞅 | 🞅 | 🞅 | 🞅 | 🞅 |
| 4. Sitting and talking on the phone. | 🞅 | 🞅 | 🞅 | 🞅 | 🞅 | 🞅 | 🞅 | 🞅 | 🞅 |
| 5. Doing paperwork or computer work (office work, emails, paying bills, etc.) | 🞅 | 🞅 | 🞅 | 🞅 | 🞅 | 🞅 | 🞅 | 🞅 | 🞅 |
| 6. Sitting reading a book or magazine. | 🞅 | 🞅 | 🞅 | 🞅 | 🞅 | 🞅 | 🞅 | 🞅 | 🞅 |
| 7. Playing a musical instrument. | 🞅 | 🞅 | 🞅 | 🞅 | 🞅 | 🞅 | 🞅 | 🞅 | 🞅 |
| 8. Doing artwork or crafts. | 🞅 | 🞅 | 🞅 | 🞅 | 🞅 | 🞅 | 🞅 | 🞅 | 🞅 |
| 9. Sitting and driving in a car, bus, or train. | 🞅 | 🞅 | 🞅 | 🞅 | 🞅 | 🞅 | 🞅 | 🞅 | 🞅 |
